# Suboptimal PAM Enables One-Pot RPA–CRISPR/Cas12a Detection of *Clostridium perfringens* in Food Samples

**DOI:** 10.3390/cimb48090867

**Published:** 2026-08-26

**Authors:** Bo Li, Xin Li, Kai Li, Shenquan Liao, Mingfei Sun, Fuqiang Huang, Haoji Zhang

**Affiliations:** 1 School of Animal Science and Technology, Foshan University, Foshan 528225, China; lb543669@163.com (B.L.);; 2 College of Veterinary Medicine, Nanjing Agricultural University, Nanjing 210095, China; 3 Institute of Animal Health, Guangdong Academy of Agricultural Sciences, Guangzhou 510640, China

**Keywords:** *Clostridium perfringens*, food, suboptimal PAM, CRISPR/Cas12a, recombinase polymerase amplification

## Abstract

The strict requirement for a canonical TTTV protospacer adjacent motif (PAM) restricts target-site selection in clustered regularly interspaced short palindromic repeats/CRISPR-associated protein 12a (CRISPR/Cas12a)-based diagnostics and limits the flexibility of assay design. Whether suboptimal PAMs can be effectively incorporated into one-pot detection systems for *Clostridium perfringens* has not been systematically examined. Here, we developed two one-pot recombinase polymerase amplification (RPA)–CRISPR/Cas12a assays targeting the *plc* and *cpe* toxin genes of *C. perfringens* and systematically evaluated 28 crRNAs spanning canonical (TTTV) and suboptimal (VTTV, TCTV) PAMs in both trans-cleavage and one-pot formats. Several suboptimal-PAM crRNAs, including crRNA 4 for *plc* (PAM: GTTG) and crRNA 7 for *cpe* (PAM: CTTA), maintained efficient detection in the one-pot system, whereas crRNA performance between the two formats was not predictable, underscoring the need for direct one-pot screening. The optimized assays achieved limits of detection of 50 copies (*plc*) and 10 copies (*cpe*), showed no cross-reactivity against four non-target bacterial species. The *plc* assay was successfully validated in pork, chicken, and beef samples spiked with *C. perfringens*, with results readable within 40 min using a simple isothermal device and naked-eye readout. The *cpe* assay was confirmed as a proof-of-concept at the genomic DNA level. These results demonstrate that suboptimal PAMs can serve as a deliberate design strategy to expand target accessibility in one-pot CRISPR/Cas12a diagnostics.

## 1. Introduction

*Clostridium perfringens* is one of the most common foodborne bacterial pathogens worldwide. Enterotoxigenic strains producing *C. perfringens* enterotoxin (CPE) are responsible for an estimated one million cases of foodborne diarrhea annually in the United States alone, with comparable disease burdens reported across Europe [1,2]. Meat and poultry products are the primary vehicles of transmission, and CPE–positive *C. perfringens* has been detected in up to 25% of retail chicken carcasses [3]. Because CPE-producing strains survive cooking and form resistant spores, food contamination often leads to outbreaks even in properly handled products [4].

*C. perfringens* is classified into types A through G based on its toxin profile [5]. The *plc* gene, encoding α-toxin (CPA), is located on the chromosome and is conserved across all *C. perfringens* strains, whereas the *cpe* gene, encoding CPE, is carried by a subset of strains and may reside on either the chromosome or a plasmid [6,7]. Detection of *plc* thus indicates the presence of *C. perfringens*, while detection of *cpe* identifies strains with enterotoxigenic potential. Rapid, on-site detection of both genes is essential for food safety surveillance and outbreak response.

Current gold-standard methods rely on bacterial culture, requiring 2 to 5 days including selective enrichment, biochemical confirmation, and serological analysis [8,9,10]. Although PCR and qPCR offer improved sensitivity and toxin typing, they depend on laboratory infrastructure [11,12]. Clustered regularly interspaced short palindromic repeats/CRISPR-associated protein 12a (CRISPR/Cas12a) has emerged as a promising alternative: upon target recognition, its trans-cleavage activity indiscriminately cleaves single-stranded DNA reporters, enabling fluorescence or lateral-flow readout at a constant temperature [13,14,15]. When combined with recombinase polymerase amplification (RPA), nucleic acid amplification and signal generation can be integrated into a single closed-tube reaction, reducing contamination risk and simplifying operational operation [16]. One-pot RPA–CRISPR/Cas12a platforms have been widely explored for detecting foodborne pathogens and antimicrobial resistance genes [17,18].

A key constraint, however, limits the flexibility of Cas12a–based assay design: the dependence on a canonical TTTV protospacer adjacent motif (PAM). Because PAM availability directly determines the number of accessible target sites, this requirement can substantially restrict crRNA selection, particularly in conserved genomic regions that lack nearby TTTV sequences. Biochemical and structural studies have established that Cas12a retains partial activity toward certain noncanonical PAMs [19,20], and suboptimal PAMs have been used to slow cis-cleavage and improve one-pot compatibility [19,21]. However, these observations have been derived primarily from isolated trans-cleavage assays or genome-editing studies. Whether suboptimal PAM tolerance is preserved under the competitive conditions of a one-pot reaction, where target amplification and Cas12a activation occur simultaneously, remains poorly understood.

**Figure 1:**
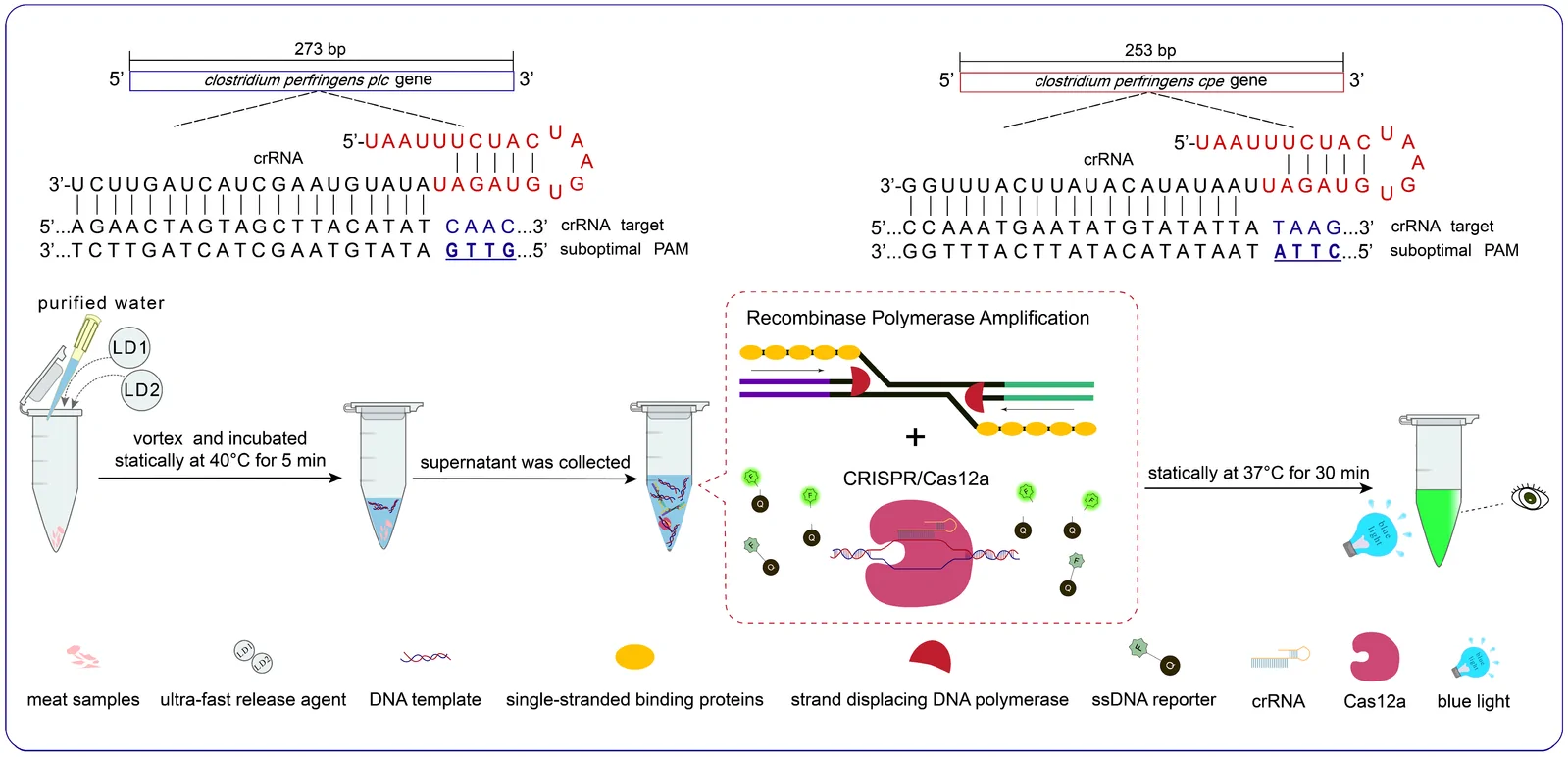
Schematic of the one-pot RPA–CRISPR/Cas12a detection workflow for *C. perfringens* with a total turnaround time of approximately 40 min. During on-site detection, a 20 mg sample is cut into small fragments and mixed with purified water, LD1 (lysis buffer), and LD2 (neutralization buffer) in an Eppendorf tube. After vortexing and incubation at 40 °C for 5 min (lysis step), the supernatant is collected as the DNA template and added directly to the one-pot reaction tube. Following incubation at 37 °C for 30 min (one-pot reaction), results are read under blue light with the naked eye. Sample handling and readout require approximately 5 min, bringing the total workflow to approximately 40 min.

In this study, we developed a one-pot RPA–CRISPR/Cas12a assay targeting the *plc* and *cpe* genes of *C. perfringens* and systematically evaluated 28 crRNAs spanning canonical (TTTV) and suboptimal (VTTV, TCTV) PAMs in both trans-cleavage and one-pot formats (Figure 1). We investigated whether suboptimal-PAM crRNAs could maintain efficient detection while expanding target-site accessibility, and validated the optimized assays using artificially contaminated food samples.

## 2. Materials and Methods

### 2.1. Reagents and Bacterial Strains

Cas12a nuclease was purchased from Guangzhou Aidi Gene Technology (Guangzhou, China). The single-stranded DNA (ssDNA) reporter (FAM-TTATT-BHQ1) and all RPA primers (Table S1) were synthesized by Sangon Biotech (Shanghai, China). The RPA kit was obtained from Amp-Future Biotech (Weifang, China). *C. perfringens* type A (CVCC52) was provided by the Institute of Animal Health, Guangdong Academy of Agricultural Sciences (Guangzhou, China). TB Green Premix Ex Taq II was purchased from Takara Biomedical Technology (Beijing, China), and the T7 High-Efficiency Transcription Kit was obtained from TransGen Biotech (Beijing, China).

### 2.2. Culture and Enumeration of C. perfringens

*C. perfringens* was streaked onto tryptose sulfite cycloserine (TSC) plates and incubated anaerobically at 37 °C for 12 h. A single black colony was isolated and placed into a fluid thioglycollate (FT) medium, which was then statically incubated at 37 °C for 8 h until it reached the logarithmic growth phase. For enumeration, the bacterial cultures were serially diluted in an FT medium. Aliquots of 100 μL from the 10^−5^, 10^−6^, and 10^−7^ dilutions were plated onto TSC plates, with three parallel controls. After incubation, the plates’ black colonies were counted, and the total colony count was calculated.

### 2.3. Nucleic Acid Preparation

The DC112-01 tissue DNA isolation mini kit (Novozymes Biotechnology Co., Ltd., Shenyang, China) was employed to rapidly extract DNA from pork, chicken, and beef. DC103-01 bacteria DNA isolation mini kit (Novozymes Biotechnology Co., Ltd., Shenyang, China) was used to rapidly extract DNA from *C. perfringens*, *Escherichia coli*, *Salmonella*, *Paeniclostridium sordellii*, and *Proteus mirabilis*. All DNA samples were stored at −20 °C until use. The ultra-fast release agent WLD8201 (Amp-Future Co., Ltd., Weifang, China) was used for on-site sample detection. The extraction system was optimized (Figure S1). For samples of pork, chicken, and beef, 100 μL of purified water, 32 μL LD1, and 8 μL LD2 were added to every 20 mg of minced sample, and the mixture was thoroughly homogenized and incubated statically at 40 °C for 5 min. The supernatant was collected as the DNA template.

The preparation of the standard plasmid for the *plc* gene involved extracting the genomic DNA of *C. perfringens*, followed by specific amplification with designed PCR primers (Table S1). The target fragment was recovered from the gel, ligated into the pMD18-T vector, and transformed into DH5α competent cells. After expansion and plasmid extraction, sequencing confirmed the plasmid’s accuracy before storage for future use. The standard plasmid of the *cpe* gene was obtained from Tsingke Biotechnology Co., Ltd. (Beijing, China).

### 2.4. Sequence Alignment and Target Region Selection

Prior to primer and crRNA design, an extensive sequence alignment was performed using publicly available *plc* and *cpe* gene sequences deposited in the National Center for Biotechnology Information (NCBI) database. Conserved regions within each target gene were identified to ensure broad coverage of diverse *C. perfringens* strains, while simultaneously guaranteeing that the selected sequences exhibited no significant homology to other bacterial species commonly found in food matrices. This sequence-based prescreening ensured high theoretical inclusivity and specificity before experimental validation.

### 2.5. Design and Selection of RPA Primers

RPA amplicons are typically~200 bp in length, which restricts the genomic regions available for crRNA design. To identify the best combination, two regions within the *plc* and *cpe* genes were selected for designing the RPA primers and corresponding crRNAs. The RPA primers were developed following the design principles reported by Lobato et al. (Table S1) [22]. The secondary structures of the primers were analyzed using the online tool, Multiple Primer Analyser (Thermo Fisher Scientific Inc., Waltham, MA, USA). RPA reaction: The reaction mixture consisted of 29.4 μL of Buffer A, upstream and downstream primers at a final concentration of 400 nM, 11.6 μL of DEPC water, 2.5 μL of Buffer B, and 2.5 μL of DNA template in a total volume of 50 μL. After incubation at 37 °C for 30 min, DNA was extracted using an extraction buffer (Tris-saturated phenol/chloroform/isoamyl alcohol 25:24:1). The RPA amplification was observed under UV light after agarose gel electrophoresis.

### 2.6. Design and Preparation of crRNA

We designed crRNAs to target regions within the range of the RPA primers, including both the standard PAM TTTV and the suboptimal PAMs VTTV and TCTV (Table S2). DNA templates corresponding to each crRNA were designed for crRNA synthesis, which included the T7 promoter, as well as the repeat and spacer sequences of the crRNA. The crRNAs were synthesized through in vitro transcription, followed by purification using phenol-chloroform extraction, and dissolved in DEPC water. The concentration was then quantified using a Qubit fluorometer (Thermo Fisher Scientific, Waltham, MA, USA).

### 2.7. Trans-Cleavage Reaction

The trans-cleavage reaction mixture consisted of the following components: 1 μL of Cas12a (33 nM), 1 μL of crRNA (33 nM), 3 μL of NEBuffer 2.1 (1×), and 1.2 μL of ssDNA reporter (400 nM). The concentration of the template was adjusted to 3.5 nM and the total volume was made up to 30 μL using DEPC water. The reaction mixture was incubated at ambient temperature for 20 min to facilitate the formation of the Cas12a/crRNA ribonucleoprotein (RNP), after which the template was introduced. Following thorough mixing, the reaction was conducted at 37 °C for 30 min on an Isothermal Nucleic Acid Amplification Analyzer (Amp-Future Biotech Co., Ltd., Weifang, China) with the FAM signal reading at 1 min intervals. The template concentration for the trans-cleavage assay (3.5 nM) was chosen to ensure measurable collateral activity in the absence of amplification, as the DNA substrate directly activates the Cas12a/crRNA RNP complex without signal enrichment [19].

### 2.8. One-Pot Reaction

One-pot reaction system: The reaction mixture comprised 18 μL of RPA mixture, 1 μL of Cas12a (33 nM), 1 μL of crRNA (33 nM), 3 μL of NEBuffer 2.1 (1×), 1.2 μL of ssDNA reporter (400 nM), 2 μL of buffer B, 1.8 μL of DEPC water, and 2 μL of DNA template, comprising a total volume of 30 μL. Following thorough mixing, the reaction was conducted at 37 °C for 30 min on an Isothermal Nucleic Acid Amplification Analyzer (Amp-Future Biotech Co., Ltd., Weifang, China) with the FAM signal reading at 1 min intervals. For the one-pot reactions, a substantially lower template concentration (2.34 pM) was used to mimic low–copy target conditions and to allow the RPA amplification process to generate amplicons for Cas12a detection, consistent with the methodological framework established by Lu et al. [19].

### 2.9. Spiked Sample Preparation

Pork, chicken, and beef were used as food samples to validate the method. Initially, the samples were analyzed by qPCR. Cultured *C. perfringens* (CVCC52) was added to the uncontaminated samples at final concentrations ranging from 10^6^, 10^5^, 10^4^, 10^3^, 10^2^, 10^1^, and 0 colony-forming units (CFU). Target DNA was extracted from the spiked samples using a tissue DNA isolation mini kit and an ultra-fast release agent, respectively. Detection was performed using both qPCR and the RPA–Cas12a–*plc* method.

### 2.10. The qPCR Assay

The qPCR method is based on the work of Ravinder Nagpal et al. (Table S1), each reaction mixture (20 μL) consisted of 10 μL of TB Green Premix Ex Taq II, 0.8 μL of the forward primer, 0.8 μL of the reverse primer, 6.4 μL of DEPC water, and 2 μL of DNA template. The reaction procedures were as follows: one cycle at 95 °C for 30 s, followed by 40 cycles at 95 °C for 15 s, 60 °C for 30 s, and 72 °C for 30 s [11].

## 3. Results

### 3.1. Selection of RPA Primers Targeting plc and cpe Genes

**Figure 2:**
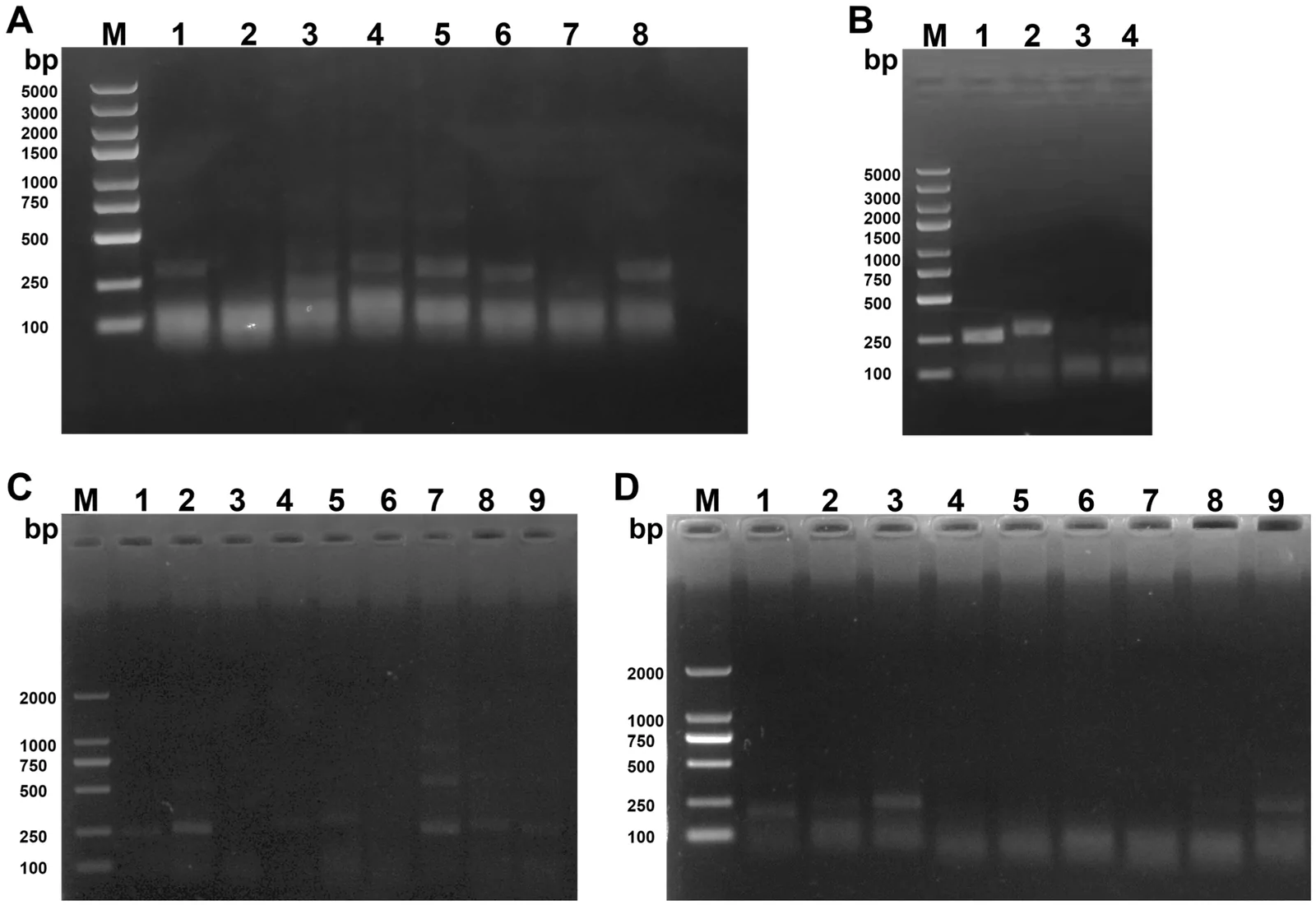
Screening of RPA primers. M: DNA Marker. (**A**) lanes 1–8 represent the region 1 RPA primers of *plc* gene: F1/R1, F1/R2, F2/R2, F2/R3, F2/R1, F3/R1, F3/R4 and F4/R5. (**B**) lanes 1–4 represent the region 2 RPA primers of *plc* gene: F5/R7, F5/R6, F6/R6, and F6/R7. (**C**) lanes 1–9 represent the region 1 RPA primers of *cpe* gene: F1/R1, F1/R2, F1/R3, F2/R1, F2/R2, F2/R3, F3/R1, F3/R2, F3/R3. (**D**) lanes 1–9 represent the region 2 RPA primers of *cpe* gene: F4/R4, F4/R5, F4/R6, F5/R4, F5/R5, F5/R6, F6/R4, F6/R5, F6/R6.

To establish a sensitive detection platform, candidate RPA primers targeting conserved regions of the *plc* and *cpe* genes were screened. Among the tested primer combinations, F5/R7 targeting the *plc* gene produced strong amplification signals (Figure 2A,B), whereas F1/R2 showed superior amplification efficiency for the *cpe* gene (Figure 2C,D). These primer pairs were therefore selected for subsequent CRISPR/Cas12a assay development (Table 1).

### 3.2. Identification of crRNAs Compatible with Suboptimal PAMs

**Figure 3:**
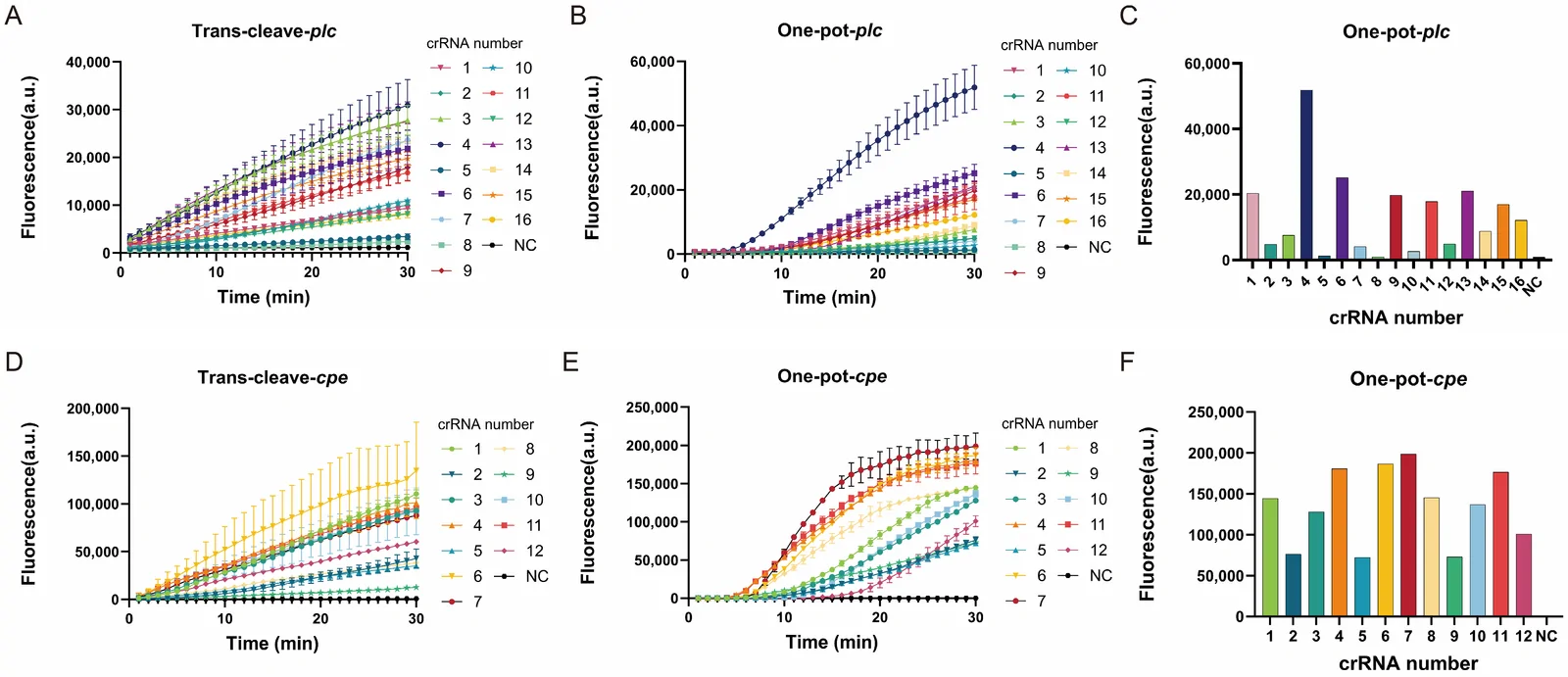
Screening of crRNA Candidates. (**A**,**D**) The fluorescence curves derived from trans-cleavage assays involving 16 crRNAs targeting the *plc* gene and 12 crRNAs targeting the *cpe* gene, respectively. (**B**) Fluorescence curves from one-pot reactions combining 16 crRNAs targeting the *plc* gene with corresponding RPA primers. (**C**) Endpoint fluorescence values for one-pot reactions combining 16 crRNAs targeting the *plc* gene with RPA primers, displayed as a bar graph. (**E**) Fluorescence curves from one-pot reactions combining 12 crRNAs targeting the *cpe* gene with corresponding RPA primers. (**F**) Endpoint fluorescence values for one-pot reactions combining 12 crRNAs targeting the *cpe* gene with RPA primers, displayed as a bar graph. Each condition was tested in one experiment with two parallel reactions (*n* = 2 per group). Data are expressed as mean ± SD.

A total of 16 crRNAs targeting the *plc* gene and 12 targeting the *cpe* gene were prepared, spanning three PAM types: TTTV (canonical), VTTV, and TCTV (suboptimal). All crRNAs activated trans-cleavage activity in the presence of 3.5 nM target DNA, including those targeting suboptimal PAMs (Figure 3A,D). Notably, crRNAs 3 (PAM: CTTG) and 4 (PAM: GTTG) for *plc*, and crRNAs 4 (PAM: TCTC) and 6 (PAM: TCTA) for *cpe*, showed particularly strong trans-cleavage signals.

When combined with RPA primers in one-pot reactions at 2.34 pM template, however, crRNA performance diverged markedly from the trans-cleavage results. For *plc*, crRNA 4 (PAM: GTTG) produced the earliest fluorescence signal (4 min) and the highest endpoint fluorescence (Figure 3B,C). For *cpe*, crRNA 7 (PAM: CTTA) initiated fluorescence at approximately 6 min and, despite a slightly later onset than crRNAs 4 (PAM: TCTC) and 11 (PAM: TTTA), exhibited the strongest signal growth and the highest endpoint fluorescence (Figure 3E,F).

**Figure 4:**
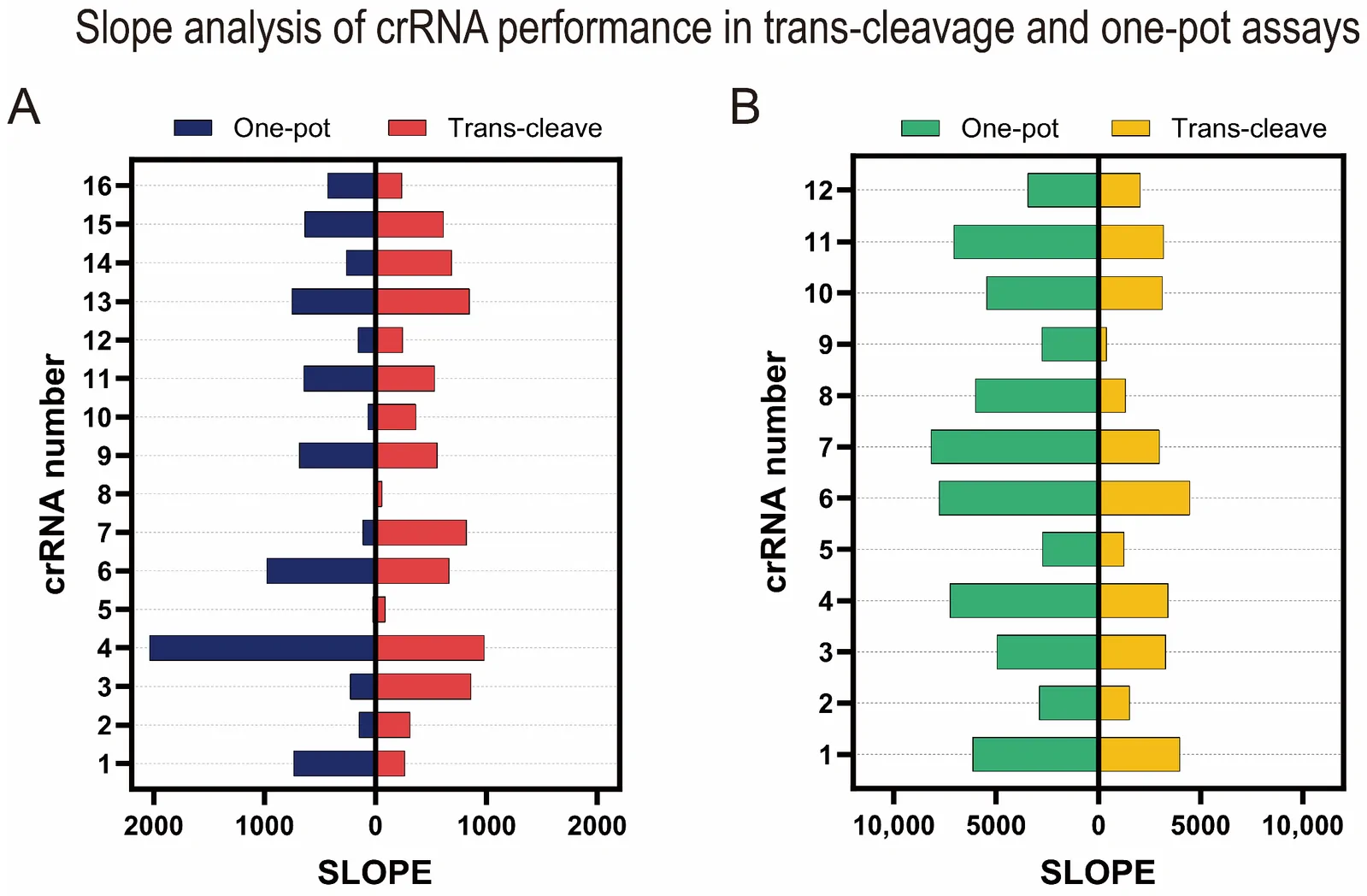
(**A**) Slope values representing the rate of fluorescence increase for each *plc* gene crRNA in trans-cleavage and one-pot assays. (**B**) Slope values representing the rate of fluorescence increase for each *cpe* gene crRNA in trans-cleavage and one-pot assays.

Comparing SLOPE values between trans-cleavage and one-pot assays confirmed that crRNAs with the highest trans-cleavage efficiency generally showed reduced performance in one-pot reactions, as expected (Figure 4A,B). Several suboptimal-PAM crRNAs, however, maintained high efficiency in both formats, crRNAs 4 (PAM: GTTG) and 13 (PAM: TTTC) for *plc*, and crRNA 6 (PAM: TCTA) and crRNA 4 (PAM: TCTC) for *cpe*. These results underscore the importance of empirical screening rather than relying solely on trans-cleavage data for crRNA selection.

### 3.3. Evaluation of Amplicon Generation in the One-Pot Reaction

**Figure 5:**
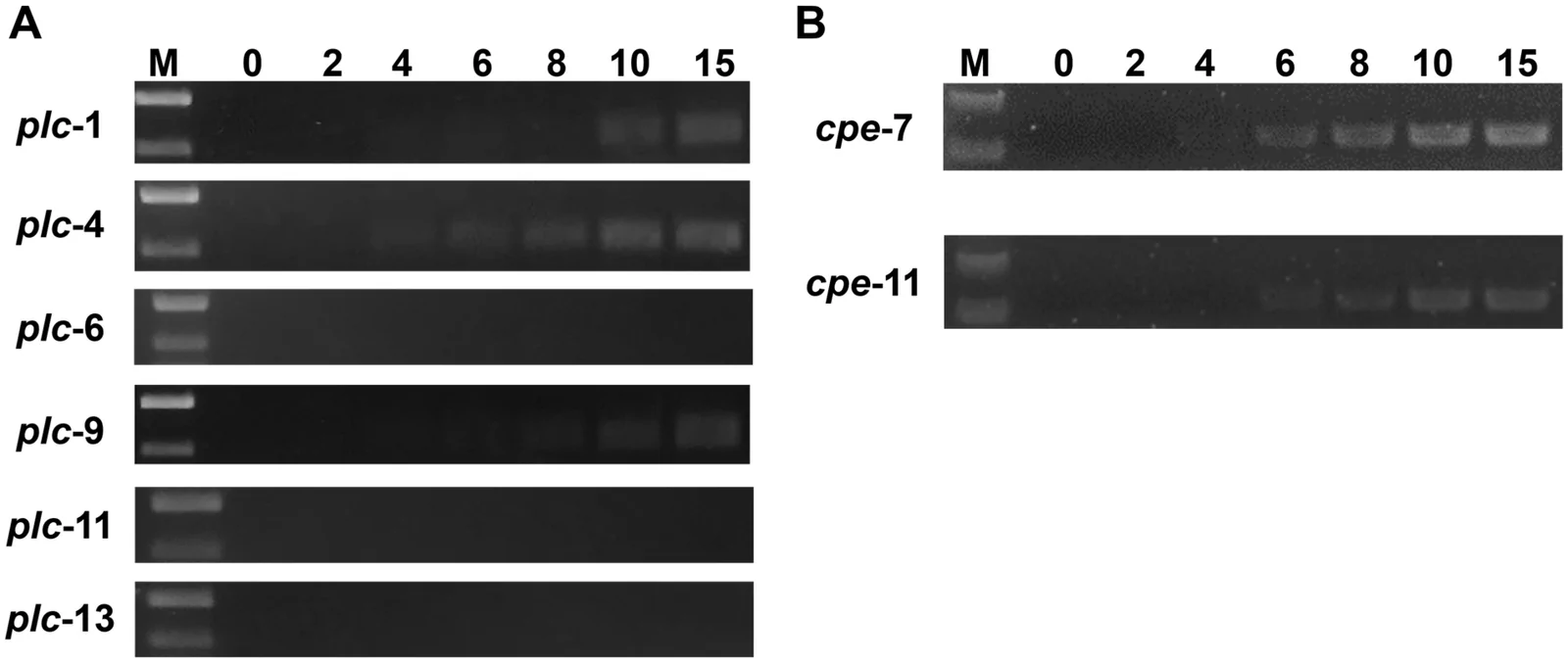
Monitoring of amplicon generation in the one-pot reaction. M: DNA Marker. 0–15: The production of amplicons was observed at 0, 2, 4, 6, 8, 10, and 15 min after the initiation of the reaction. (**A**) Comparison of amplicon production for crRNAs 1, 4, 6, 9, 11, and 13 targeting the *plc* gene in the one-pot reaction. (**B**) Comparison of amplicon production for crRNAs 7 and 11 targeting the *cpe* gene in the one-pot reaction.

To further validate the selected crRNAs, amplicon production was monitored at 0, 2, 4, 6, 8, 10, and 15 min during one-pot reactions. For the *plc* gene, the combination of crRNA 4 and primers F5/R7 yielded a visible target band at 4 min (Figure 5A). For *cpe*, crRNA 7 combined with primers F1/R2 similarly produced the target band at 4 min (Figure 5B). These combinations were therefore adopted for the final RPA–Cas12a–*plc* and RPA–Cas12a–*cpe* assays, respectively (Table 1).

### 3.4. Sensitivity of RPA-Cas12a

**Figure 6:**
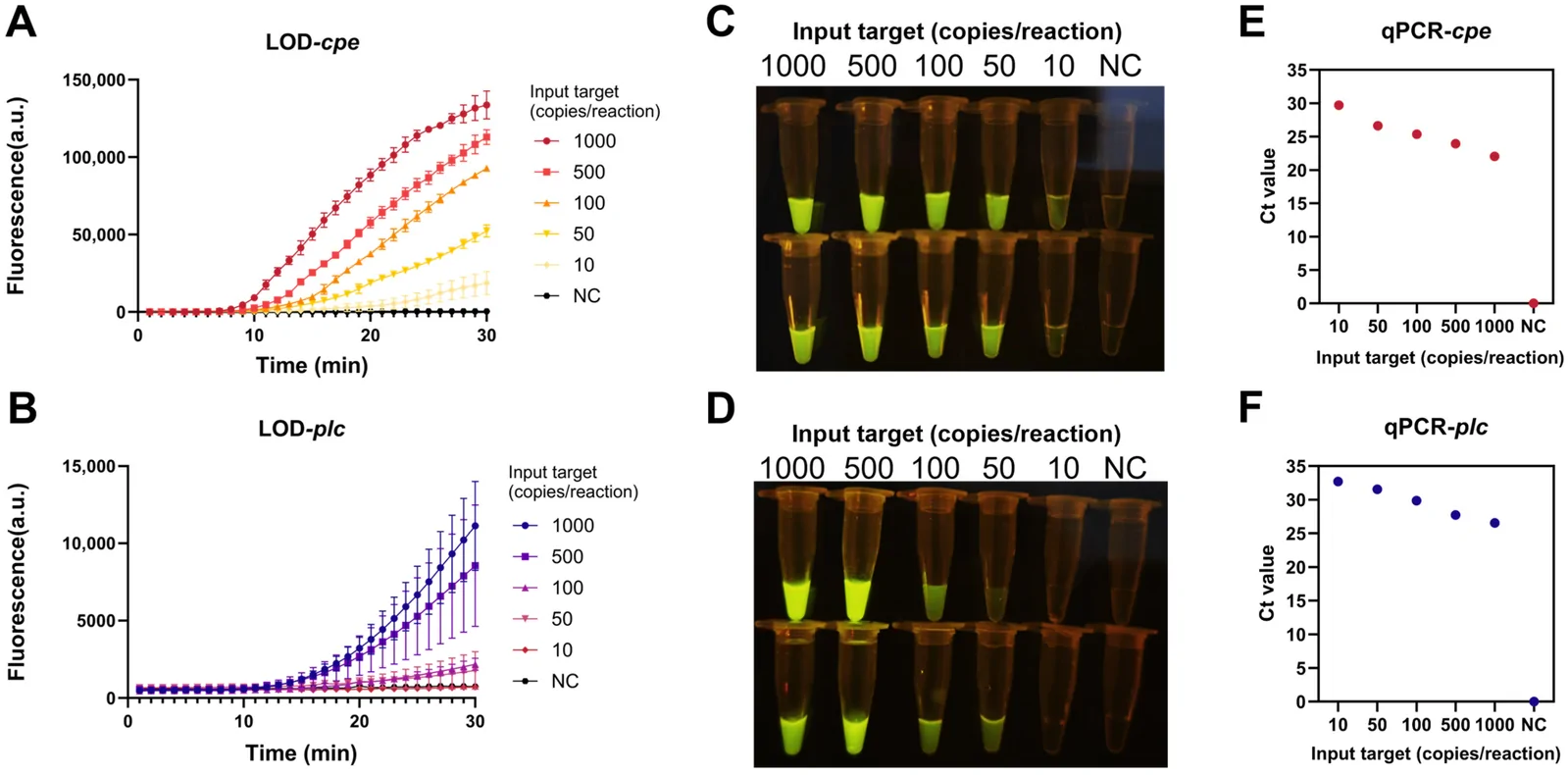
Sensitivity determination of the RPA–Cas12a detection method. (**A**,**C**) Results of plasmid standards for *plc* detection using the RPA–Cas12a–*plc* detection method. (**B**,**D**) Results of plasmid standards for *cpe* detection using the RPA–Cas12a–*cpe* detection method. (**E**) qPCR detection of *cpe* gene templates ranging from 1000 to 0 copies. (**F**) qPCR detection of *plc* gene templates ranging from 1000 to 0 copies. Each concentration was tested in one experiment with two parallel reactions (*n* = 2 per group). Data are expressed as mean ± SD.

**Figure 7:**
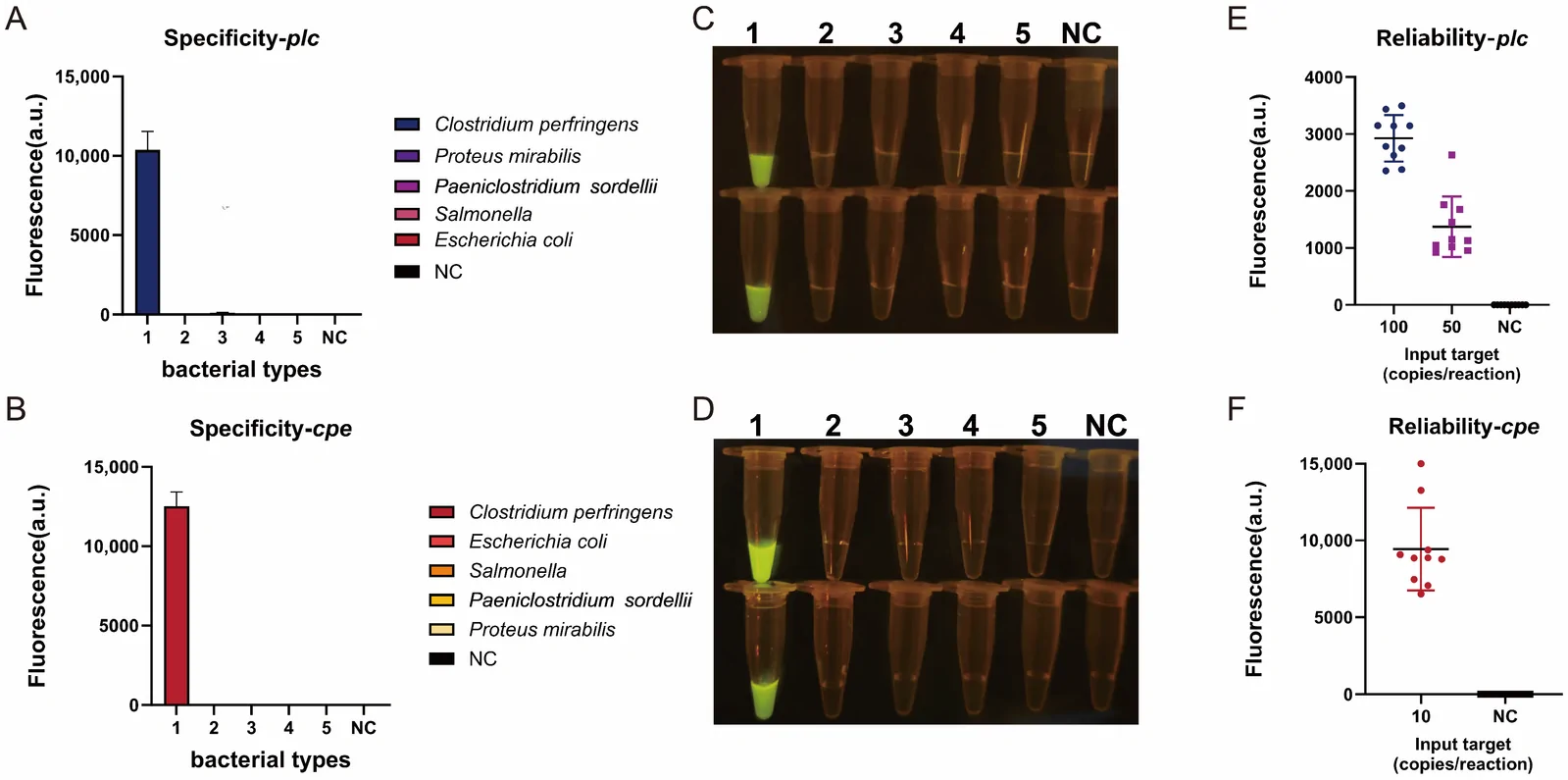
Verification of the specificity and reliability of the RPA–Cas12a detection method. (**A**,**C**) Results of *C. perfringens*, *E. coli*, *Salmonella*, *P. sordellii*, and *P. mirabilis* detection using the RPA–Cas12a–*plc* detection method; (**B**,**D**): Results of *C. perfringens*, *E. coli*, *Salmonella*, *P. sordellii* and *P. mirabilis* detection using the RPA–Cas12a–*cpe* detection method; (**E**) Reliability analysis results of the RPA–Cas12a–*plc* detection method; (**F**) Reliability analysis results of the RPA–Cas12a–*cpe* detection method. For specificity testing (**A**–**D**), each condition was tested in one experiment with two parallel reactions (*n* = 2 per group). Data are expressed as mean ± SD. For reliability testing (**E**,**F**), each experiment was performed with two parallel reactions and repeated five times independently (*n* = 10 per group). Data are expressed as mean ± SD with individual data points overlaid.

To validate the sensitivity of the established detection method, qPCR and RPA–Cas12a were used to detect plasmid standards with 1000, 500, 100, 50, 10, and 0 copies. The results showed that the lower limit of detection (LOD) for the RPA–Cas12a–*cpe* method was 10 copies, comparable to that of qPCR, which detected 10 copies with a Ct value of 29.7 ± 0.2 (Figure 6A,E). The LOD for the RPA–Cas12a–*plc* method was 50 copies, slightly lower than that of qPCR, which still detected 10 copies with a cycle threshold (Ct) value of 32.69 (Figure 6B,F). The LOD was defined as the lowest template concentration that consistently produced detectable fluorescence signals in all parallel reactions. Both detection methods exhibited observable fluorescence in the reaction tubes at their respective detection thresholds (Figure 6C,D). The reproducibility of the LODs was further validated in five independent experiments (biological replicates, *n* = 5), each performed with two parallel reactions, using templates at the LOD concentrations (10 copies for *cpe*; 50 and 100 copies for *plc*). Positive signals were consistently detectable across all replicates (Figure 7E,F), confirming the reproducibility of the reported LODs.

### 3.5. Reliability and Specificity of RPA–Cas12a

To validate the reliability of the RPA–Cas12a detection method, templates at the detection limit quantity were tested using five biological replicates, each performed with two parallel reactions. The results showed that positive signals were consistently detectable across all replicates (Figure 7E,F). To verify the specificity of the RPA–Cas12a detection method, *C. perfringens*, *E*. *coli*, *Salmonella*, *P*. *sordellii*, and *P*. *mirabilis* were tested. Only reaction tubes containing *C. perfringens* showed fluorescence in both detection methods, indicating good specificity against the bacterial species tested (Figure 7A–D).

### 3.6. Analysis of Spiked Food Samples

**Figure 8:**
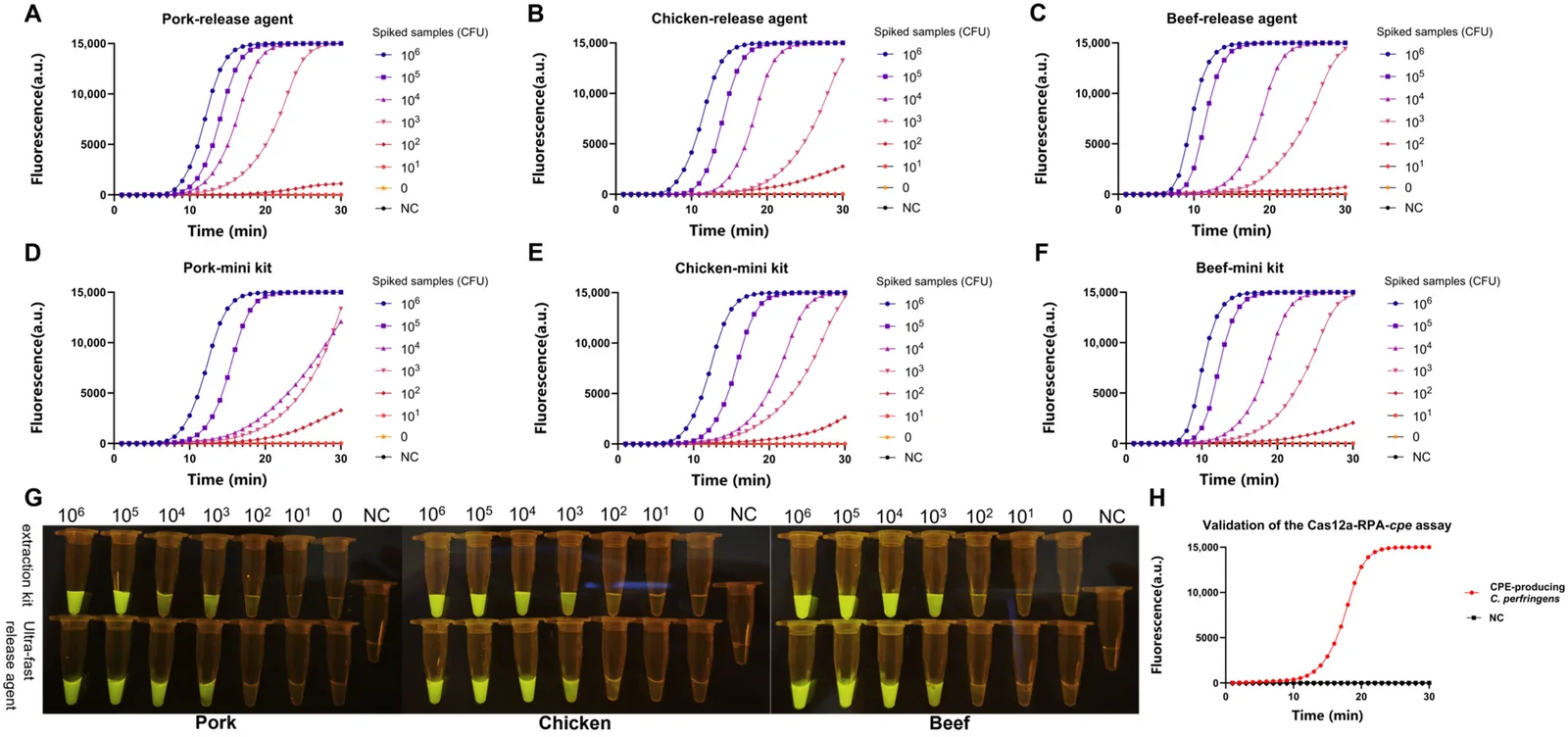
Validation of the utility of the RPA–Cas12a method using spiked samples. (**A**–**C**) Detection of target DNA from pork, chicken, and beef samples, extracted using the ultra-fast release agent, with the RPA–Cas12a–*plc* method. (**D**–**F**) Detection of target DNA from pork, chicken, and beef samples, extracted using the tissue DNA isolation mini kit, with the RPA-Cas12a-*plc* method. (**G**) Detection results of all spiked samples under blue light. (**H**) Detection of *C. perfringens* genomes carrying the *cpe* toxin gene using the RPA–Cas12a–*cpe* method. Each spiking condition was tested once without replicates as a preliminary validation of matrix compatibility. Data are shown as representative results from single measurements.

Pork, chicken, and beef samples spiked with *C. perfringens* were selected to validate the efficacy of the RPA–Cas12a–*plc* detection method. DNA was extracted from samples containing 10^6^, 10^5^, 10^4^, 10^3^, 10^2^, 10^1^, and 0 CFU of *C. perfringens* using either a tissue DNA isolation mini kit or an ultra-fast release agent. The RPA–Cas12a–*plc* method was employed to detect *C. perfringens* in the three spiked samples. In this preliminary validation using spiked food samples, the RPA–Cas12a–*plc* method detected *C. perfringens* at spiking levels down to 10^2^ CFU in all three matrices when using either DNA extraction method (Figure 8G). In samples spiked with 10^3^–10^6^ CFU, DNA extracted using the ultra-fast release agent showed earlier peak times compared to column-based extraction (Figure 8A–F). However, at 10^2^ CFU, the ultra-fast release agent exhibited variable performance across matrices, with reliable detection observed in chicken samples but not consistently in pork and beef (Figure 8A–F). Given that these experiments were performed as single measurements, these observations should be considered preliminary. Independent replicate experiments are required to validate these findings. Due to the current unavailability of CPE–producing *C. perfringens* isolates in our laboratory, the RPA–Cas12a–*cpe* assay was validated using genomic DNA extracted from a CPE–producing strain as a proof-of-concept confirmation. The assay produced a clear positive fluorescence signal under the established conditions (Figure 8H), confirming the functionality of the primer and crRNA set for the *cpe* target.

## 4. Discussion

Isothermal methods such as loop–mediated isothermal amplification (LAMP) and RPA have moved toward on-site detection of *C. perfringens*, yet achieving both high sensitivity and sequence specificity in a single, streamlined workflow remains a challenge [9,22,23].The one-pot RPA–CRISPR/Cas12a format addresses this challenge but introduces a critical design constraint: RPA and Cas12a compete for the same DNA template. Cas12a–mediated cis–cleavage of target molecules during early reaction stages depletes the substrate needed for RPA amplification, resulting in attenuated signals [19]. Suboptimal PAMs slow the initial cis-cleavage rate, allowing amplicon accumulation before trans-cleavage signal generation [19,21]. Consistent with this principle, most crRNAs that showed high efficiency in trans-cleavage assays performed poorly in one-pot reactions (Figure 4A,B). Importantly, several suboptimal-PAM crRNAs, including crRNA 4 for *plc* and crRNA 7 for *cpe*, maintained high efficiency in both formats, suggesting that PAM identity alone may not fully determine one-pot performance. The structural features of individual crRNAs, particularly their spacer sequences, may also contribute to RNP complex formation and Cas12a activity [24]; however, further investigation is warranted to clarify the underlying mechanisms. This observation highlights the need for systematic empirical screening when developing one-pot assays, rather than relying on trans-cleavage results alone. It should be noted that the trans-cleavage and one-pot assays in this study were performed with substantially different template concentrations (3.5 nM for trans-cleavage versus 2.34 pM for one-pot reactions), following the methodological precedent established by Lu et al. for suboptimal PAM-based one-pot CRISPR/Cas12a detection [19]. This concentration difference reflects the distinct purposes of the two assays: the trans-cleavage assay, performed without amplification, requires a relatively high sub-strate concentration to evaluate the intrinsic enzymatic activity of each crRNA, whereas the one-pot assay, which integrates RPA amplification, employs a low template concentration to simulate realistic low-copy detection scenarios and to assess the functional compatibility of each crRNA with the amplification process. Consequently, direct quantitative comparisons of crRNA performance between the two formats should be interpreted with this concentration gap in mind. Nevertheless, the qualitative conclusion that crRNA performance ranks differently between the two formats—and that trans-cleavage data alone cannot reliably predict one-pot performance—remains valid, as it is based on the observed divergence in performance order rather than on absolute quantitative metrics.

Both assays developed here showed good specificity against the four non-target bacterial species tested, with no cross-reactivity observed. The experimental panel included *E. coli*, *Salmonella*, *P. sordellii*, and *P. mirabilis*, which represent the most relevant non-target bacteria commonly encountered in meat products. We acknowledge that this panel is not exhaustive. During the assay design phase, we performed extensive in silico sequence analysis to ensure both sequence conservation within the target genes and the absence of homology to non-target taxa. This analysis can serve as a complementary line of evidence supporting assay specificity. While this computational analysis supports target specificity, it does not replace experimental validation with a broader range of bacterial strains. In terms of sensitivity, the assays achieved LODs of 50 copies for *plc* and 10 copies for *cpe*. The *cpe* assay matched the sensitivity of qPCR, while the *plc* assay was slightly less sensitive. When combined with the ultra-fast DNA release reagent, the entire workflow from sample preparation to visual readout was completed within 40 min—comprising 5 min for sample lysis at 40 °C, 30 min for the one-pot reaction at 37 °C, and approximately 5 min for sample handling and visual readout under blue light—using only a simple isothermal device, with results visible to the naked eye under blue light. In spiked food samples, the release reagent performed comparably to column-based extraction at spiking levels of 10^3^ CFU and above; however, its reliability decreased at 10^2^ CFU for certain matrices (pork and beef), likely due to incomplete DNA release and/or the presence of RPA inhibitors.

Several one-pot RPA–CRISPR/Cas12a systems have been developed for foodborne pathogen detection. For instance, a light-controlled one-pot system for MRSA detection achieved LODs of 6.3 CFU/mL (conventional crRNA) and 34.7 CFU/mL (tailed crRNA) using a photocleavable linker-based strategy [25]. A one-pot assay for Cronobacter sakazakii reported an LOD of 1.43 copies/μL for target DNA and 6 CFU/mL for pure culture using a pipette tip-in-tube strategy [18]. More recently, a multiplex RPA–CRISPR/Cas12a platform for *C. perfringens* toxinotyping achieved a detection limit of ≤10 copies/μL for six toxin genes in two reaction tubes, with a total assay time of approximately 50 min [26]. Compared with these platforms, our assay offers several distinctive advantages. First, in terms of target selection, our systematic evaluation of 28 crRNAs spanning canonical and suboptimal PAMs—combined with rigorous in silico sequence screening targeting conserved regions of the *plc* and *cpe* genes—provides a rational design framework that expands target accessibility beyond the constraints of canonical PAM dependence. Second, regarding analytical sensitivity, our assays achieved LODs of 50 copies (*plc*) and 10 copies (*cpe*), which are comparable to or better than those of previously reported one-pot systems, and the *cpe* assay matched the sensitivity of qPCR. Third, with respect to assay time, the entire workflow from sample preparation to visual readout was completed within 40 min using only a simple isothermal device, which is faster than the 50 min multiplex platform and the 1 h Cp-MRC12a assay, while remaining competitive with the 30 min Senecavirus A assay. Fourth, in terms of operational simplicity, our one-pot format, combined with an ultra-fast DNA release agent, eliminates the need for column-based extraction, thermal cyclers, or specialized instrumentation, making it particularly suitable for resource-limited settings.

Several aspects of the CRISPR/Cas12a-coupled isothermal amplification assay could be further optimized. First, the addition of glycerol to the reaction system has been demonstrated to markedly enhance detection sensitivity [27]; however, the optimal glycerol proportion requires further validation. Second, at low template concentrations, fluorescence signals may be too weak for naked-eye detection, necessitating a portable fluorescence reader; incorporating a lateral-flow readout could address this limitation. Third, the current assays detect *plc* and *cpe* in separate reactions. Multiplex RPA–CRISPR/Cas12a platforms, currently under development for other applications [28], would enable simultaneous toxin typing and further streamline the detection workflow. It should be acknowledged that all validation experiments in this study were performed using artificially contaminated food samples. While spiking experiments under controlled conditions are widely accepted for initial assay development and allow reliable determination of analytical sensitivity and matrix compatibility, they may not fully recapitulate the complexity of naturally contaminated specimens, which often harbor diverse background microflora, variable levels of co-extracted PCR inhibitors, and heterogeneous distribution of target pathogens. These factors could influence the diagnostic accuracy and robustness of the assay in routine food safety surveillance. That said, we acknowledge that the spiked food sample experiments (Figure 8) were performed as single measurements without replicates. Therefore, these results should be considered preliminary, and additional experiments with independent replicates would be needed to confirm the detection performance in different food matrices and to draw more robust comparisons between the two DNA extraction methods. We regard this as an important direction for future work. Furthermore, unlike the *plc* assay, which was validated in spiked pork, chicken, and beef samples, the *cpe* assay was evaluated only using genomic DNA from a CPE–producing strain, as such isolates were not available to us at the time of this study. This proof-of-concept confirmation demonstrates the functionality of the *cpe*-targeting primer and crRNA set; however, further validation using CPE–producing strains spiked into representative food matrices would be desirable before the *cpe* assay can be recommended for routine application. Nevertheless, the present data provide a solid proof-of-concept that suboptimal-PAM crRNAs can be effectively integrated into a one-pot RPA–CRISPR/Cas12a format. Ideally, future evaluation using naturally contaminated retail meat products or field isolates of *C. perfringens*, if available, would further strengthen the practical applicability of the method.

## 5. Conclusions

In this study, we developed two one-pot RPA–CRISPR/Cas12a assays for the rapid detection of *C. perfringens* toxin genes in food. By employing crRNAs that target suboptimal PAMs, we achieved compatible coupling of RPA amplification and Cas12a detection within a single reaction. Both assays showed good specificity against the tested non-target bacterial species and achieved LODs of 50 copies for *plc* and 10 copies for *cpe*. The *plc* assay was successfully validated in pork, chicken, and beef samples spiked with *C. perfringens*, supporting its applicability for routine food matrix testing. The *cpe* assay was confirmed as a proof-of-concept at the genomic DNA level; however, further validation in spiked food samples is needed before it can be recommended for enterotoxigenic strain identification. Independent replicate validation in food matrices remains a priority for future work. Overall, these results indicate that the developed assays show promise for on-site food safety testing in resource-limited settings.

## 6. Patents

A Chinese patent application related to this work has been filed (CN118581241 A).

## Figures and Tables

**Table 1:** Sequences of the final selected RPA primers and crRNAs.

Target Gene	Component	Identifier	Sequence (5′→3′)	PAM
*plc*	RPA forward primer	F5	CTAGCATGAGTCATAGTTGGGATGATTGG	/
	RPA reverse primer	R7	AATCATTTCCTGGGTTGTCCATTTCCCATTC	/
	crRNA	crRNA 4	AUAUGUAAGCUACUAGUUCU	GTTG
*cpe*	RPA forward primer	F1	CAGTTGGATTTACTTCTGAATTTATACAAGC	/
	RPA reverse primer	R2	CCTTGATCAATATTTCCTAAGCTATCTGC	/
	crRNA	crRNA 7	UAAUAUACAUAUUCAUUUGG	CTTA

## Data Availability

The original contributions presented in this study are included in the article/supplementary material. Further inquiries can be directed to the corresponding author(s).

## Supplementary Materials

The following supporting information can be downloaded at: https://www.mdpi.com/article/10.3390/cimb48090867/s1.
