# Supplementary material for: Suboptimal PAM Enables One-Pot RPA–CRISPR/Cas12a Detection of Clostridium perfringens in Food Samples
Source: Curr Issues Mol Biol. 2026 Aug 26;48(9):867. doi: 10.3390/cimb48090867 (PMC13605544; doi:10.3390/cimb48090867)
Supplement: Supplementary file 1 [file cimb-48-00867-s001.zip › cimb-4470126-supplementary.pdf]

Table S1. The primer sequences employed in this study.

**RPA primer region 1-*plc***	
F1	CTTAGAGATTTTAAAAGAGAACATGCATGAG
F2	CATGCATGAGCTTCAATTAGGTTCTAC
F3	CAGATTATGATAAGAACGCCTATGATC
F4	GATAAGAACGCCTATGATCTATATCAAGATC
R1	CTCAAACTTAACATGTCCTGCGCTATCAACG
R2	AATCCTCATTAGTTTTGCAACCTGCTGTG
R3	CTGTTCTTTTCTTTCCTCTGCAAAAGTCTC
R4	CCTCATTAGTTTTGCAACCTGCTGTGTTTA
R5	TCTGCAAAAGTCTCAAACTTAACATGTC
**RPA primer region 2-*plc***	
F5	**CTAGCATGAGTCATAGTTGGGATGATTGG**
F6	CATAGTTGGGATGATTGGGACTATGCAGCA
R6	TTGAAAGTATAAGTATCTTTACTTCCAGTC
R7	**AATCATTTCCTGGGTTGTCCATTTCCCATTC**
**RPA primer region 1-*cpe***	
F1	**CAGTTGGATTTACTTCTGAATTTATACAAGC**
F2	CCTAATGAAACAGGTACCTTTAGCCAATC
F3	CAGGTACCTTTAGCCAATCATTAACTAAATC
R1	CTGCAGATGTTTTACTAAGCCATATTCCTG
R2	**CCTTGATCAATATTTCCTAAGCTATCTGC**
R3	CCTAAGCTATCTGCAGATGTTTTACTAAGCC
**RPA primer region 2-*cpe***	
F4	TCCAGCTGGTAATTTATATGATTGGCGTTC
F5	AACTGATGCATTAAACTCAAATCCAGCTGG
F6	GCTGCTGCTACAGAAAGATTAAATTTAACTG
R4	CCATCACCTAAGGACTGTTCTAATTTCACTAG

R5	TCCAGCATCTAAGCTTATATCAACATAATG
R6	TTACAAGAACATATTGTCCAGCATCTAAGC
**qPCR-*plc***	
F	CCGTTGATAGCGCAGGACA
R	CCCAACTATGACTCATGCTAGCA
**qPCR-*cpe***	
F	GGTTCATTAATTGAAACTGGTG
R	AACGCCAATCATATAAATTACCAG
**PCR primers-*plc* gene standard plasmid**	
F	TGGGATGGAAAAATTGATGGAACA
R	TTTTATATTATAAGTTGAATTTCCTGAA

The sequences of the final selected RPA primers are highlighted in bold in Table S1 and also listed in Table 1 of the main text.

Table S2. The study presents the PAM, sequence, and structural diagram of the crRNA

***plc* gene**			
**number**	**PAM**	**sequences**	**structure**
1	ATTC	GGGUAAUUUCUACUAAGUGUAGAUUUUCCA ACUGAUGGAUCAUU	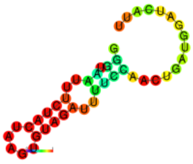
2	ATTC	GGGUAAUUUCUACUAAGUGUAGAUUAUACC UGACACAGGGGAAU	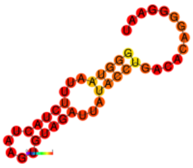
3	CTTG	GGGUAAUUUCUACUAAGUGUAGAUAGUUU UUCCAUCCUUUGUUU	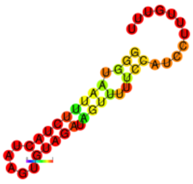
4	**GTTG**	**GGGUAAUUUCUACUAAGUGUAGAUAUAUG UAAGCUACUAGUUCU**	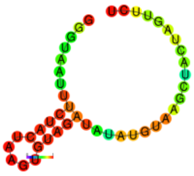

5	ATTC	GGGUAAUUUCUACUAAGUGUAGAUUUACAC<br>GAUGUAUCAGAGGG	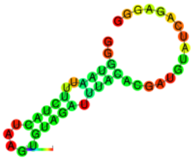
6	CTTA	GGGUAAUUUCUACUAAGUGUAGAUACAUG<br>UCCUGCGCUAUCAAC	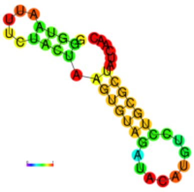
7	TCTA	GGGUAAUUUCUACUAAGUGUAGAUUAUCA<br>AGAUCAUUUCUGGGA	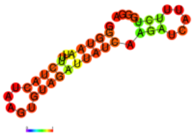
8	TCTA	GGGUAAUUUCUACUAAGUGUAGAUUACCUG<br>ACACAGGGGAAUCA	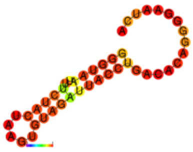
9	TCTG	GGGUAAUUUCUACUAAGUGUAGAUUUCCAG<br>CAUCUUUUUCACCA	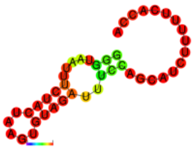
10	TCTA	GGGUAAUUUCUACUAAGUGUAGAUUCUUGG<br>AGAGGCUAUGCACU	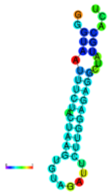
11	TCTG	GGGUAAUUUCUACUAAGUGUAGAUAUACA<br>UCGUGUAAGAAUCUA	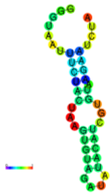
12	TTTG	GGGUAAUUUCUACUAAGUGUAGAUUGAUU<br>CCCCUGUGUCAGGUA	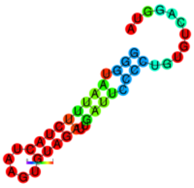

13	TTTC	GGGUAAUUUCUACUAAGUGUAGAUCUCUUU GCCAUUCAUAUCUA	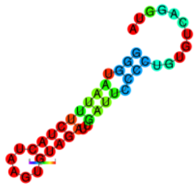
14	TTTA	GGGUAAUUUCUACUAAGUGUAGAUUAGUU UCCUCUUUGCCAUUC	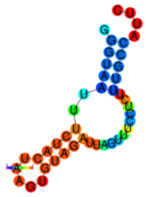
15	TTTA	GGGUAAUUUCUACUAAGUGUAGAUGCUUA UUCUAUACCUGACAC	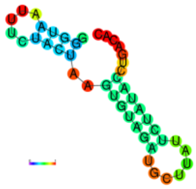
16	TTTA	GGGUAAUUUCUACUAAGUGUAGAUUAGAU UCUUACACGAUGUAU	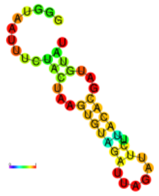
***cpe* gene**			
1	CTTC	GGGUAAUUUCUACUAAGUGUAGAUUGAAU UUAUACAAGCAUCUG	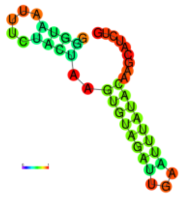
2	TCTG	GGGUAAUUUCUACUAAGUGUAGAUUAGAA UAUGGAUUUGGAAUA	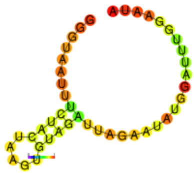
3	TCTA	GGGUAAUUUCUACUAAGUGUAGAUUUGUA UUUUGUUCUCCUAUA	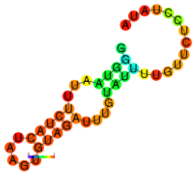
4	TCTC	GGGUAAUUUCUACUAAGUGUAGAUAUGGU AAUAUCUCUGAUGAU	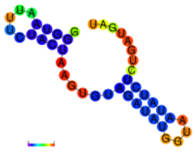

5	ATTA	GGGUAAUUUCUACUAAGUGUAGAUGAAUU UCUCAUGGUAAUAUC	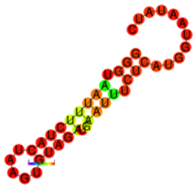
6	TCTA	GGGUAAUUUCUACUAAGUGUAGAUAUAGC UUGAUACUUUCUAUA	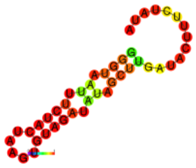
7	**CTTA**	**GGGUAAUUUCUACUAAGUGUAGAUUAAUA UACAUAUUCAUUUGG**	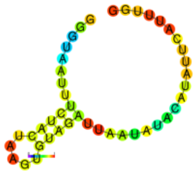
8	TTTA	GGGUAAUUUCUACUAAGUGUAGAUUAUGA UUGGCGUUCUUCUAA	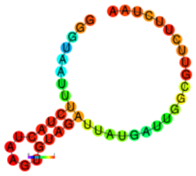
9	TTTG	GGGUAAUUUCUACUAAGUGUAGAUAGUCCA AGGGUAUGAGUUAG	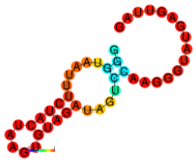
10	TTTA	GGGUAAUUUCUACUAAGUGUAGAUCACUUA ACAAUUACAGCUAC	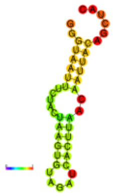
11	TTTA	GGGUAAUUUCUACUAAGUGUAGAUUUCAA AUAAUUUUAAUAAUC	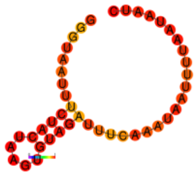
12	TTTG	GGGUAAUUUCUACUAAGUGUAGAUUCCAGU AGCUGUAAUUGUUA	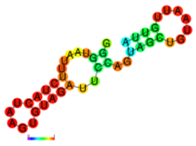

The sequences of the final selected crRNAs (spacer and PAM) are highlighted in bold in Table S2 and also listed in Table 1 of the main text.

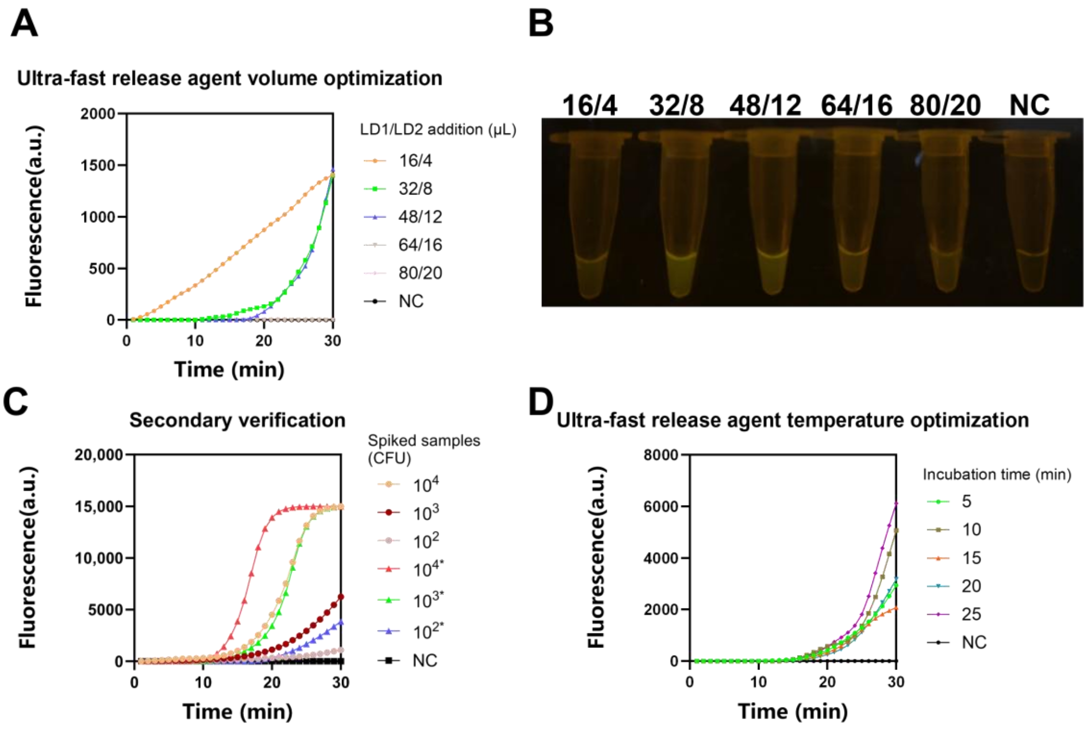

Figure S1. Optimization of the ultra-fast release agent addition. The ultra-fast release agent can extract DNA from homogenized tissue supernatant using 16 μL of LD-1 and 4 μL of LD-2. This reagent was used in this study to directly extract DNA from finely chopped meat tissue. To 20 mg of muscle tissue, 100 μL of purified water, 32 μL of LD-1, and 8 μL of LD-2 were added, followed by incubation at 40°C for 5 min. The supernatant was used as the target DNA for detection. A: The DNA samples, to which 10^2 CFU of *C. perfringens* was added, were extracted using varying volumes of LD-1/LD-2. A more significant increase in fluorescence signal was observed when the volumes of LD-1 and LD-2 were doubled and tripled. B: After incubation in a 37°C metal bath for an additional 10 min, the reaction tubes with 32/8 and 48/12 demonstrated brighter fluorescence. C: Samples containing 10^4, 10^3, and 10^2 CFU of *C. perfringens* were extracted using either 16/4 or 32/8 volumes of the ultra-fast release agent. * Denotes the 32/8 LD-1/LD-2. Using 32/8 of the ultra-fast release agent for sample DNA extraction resulted in an earlier peak time and a more robust fluorescence signal in the RPA-Cas12a-*plc* reaction. D: Samples containing 10^2 CFU of *C. perfringens* were extracted with 32/8 of LD-1/LD-2, incubated for 5, 10, 15, 20, and 25 min, and then subjected to the RPA-Cas12a-*plc* reaction. Increasing the incubation time did not result in an earlier fluorescence signal.
